# High-dose nitrate therapy recovers the expression of subtypes α_1_ and β-adrenoceptors and Ang II receptors of the renal cortex in rats with myocardial infarction-induced heart failures

**DOI:** 10.1186/s12872-020-01353-z

**Published:** 2020-02-27

**Authors:** Yubo Peng, Yanfang Li, Mengmeng Chen, Junying Song, Zhili Jiang, Shutian Shi

**Affiliations:** 1 Department of Cardiology, Aviation General Hospital, Beijing, 10016 China; 2 Department of Emergency, Anzhen Hospital, Capital Medical University, Beijing, 100029 China; 3 Hong Kong University Shenzhen Hospitall, Shenzhen, 518053 China; 4 HengShui people’s Hospital, Hengshui, 053000 HeBei Province China

**Keywords:** Chronic heart failure, Long-acting nitrate, Adrenoceptors, Ang II receptors, Renal function

## Abstract

**Background:**

Few studies examined the effect of long-acting nitrates on renal function in chronic heart failure (CHF). Thus, we aimed to investigate the effect of long-acting nitrate on the expression of adrenoceptors (AR) and angiotensin II receptor (ATR) subtypes of the renal cortex, in rats with myocardial infarction-induced CHF.

**Methods:**

Rats were randomly divided into the following groups: control, sham-operated, CHF, low- and high-dose nitrate, positive drug control (olmesartan), and high-dose of long-acting nitrate + olmesartan. Ultrasound echocardiography markers were compared, and the levels of AR subtypes, AT_1_R, and AT_2_R were measured using reverse transcription-polymerase chain reaction and western blot analysis. Histopathology of the kidney was determined on hematoxylin and eosin-stained sections.

**Results:**

CHF significantly increased plasma renin activity (PRA) and angiotensin II levels, upregulated AT_1_R expression and downregulated α_1A_-, β_1_-, β_2_-AR, and AT_2_R expression compared to the sham control. High-dose nitrate or olmesartan alone, and especially in combination, decreased the levels of PRA and angiotensin II and downregulated the CHF-induced expression of AT_1_R, α_1A_-, β_1_-, and β_2_-AR, and AT_2_R. CHF resulted in significant impairment of the renal tissue, including inflammatory cells infiltration to the tubular interstitium and surrounding the renal glomerulus, and tubular necrosis, which was alleviated in all treatment groups to different degrees.

**Conclusions:**

Long-acting nitrates could reverse CHF-induced changes in AR and ATR subtypes in the kidney, and improve cardiac function to protect renal function. Compared with monotherapy, the combination of nitrates and olmesartan shows more significant benefits in regulating AR and ATR subtypes.

## Background

Chronic heart failure (CHF) is a complex clinical syndrome in which the heart’s function cannot meet the demand of the body’s healthy blood circulation. In CHF, the useful circulating blood volume is reduced to decrease the rate of renal perfusion, which in turn activates the sympathetic nervous system (SNS), leading to increased blood catecholamine levels, which stimulates the juxtaglomerular apparatus cells to secrete and release more renin to ultimately activate the renin-angiotensin system (RAS) [1–4]. Activation of the SNS and RAS have compensatory effects during the early stage of CHF. However, by the late stage, these activations will promote the development and progression of CHF, with gradual deterioration of cardiac function.

Moreover, SNS activation in CHF will have direct effects on kidney function. Elevated catecholamine acts on the adrenergic receptor (AR) of the kidney tissue, contributing to a series of conditions, including renal interstitial inflammatory cell infiltration, renal fibrosis, and renal tubular necrosis, ultimately resulting in altered renal hemodynamics. When the RAS is activated after heart failure develops, the plasma level of angiotensin II (Ang II) also increases by mediating renal vasomotor and sodium retention, as well as the proliferation, hypertrophy, and profibroblast effect of kidney mesangial cells and the extracellular matrix. There are three kinds of α_1_-AR subtypes in the kidney tissue, within which α_1A_-AR and α_1D_-AR mainly regulate renal vasoconstriction, while α_1B_-AR mediates the proliferation and hypertrophy of vascular smooth muscle cells, and together with α_1A_-AR promotes sodium and water reabsorption in the proximal tubules [5–7].

Nitrates have beneficial effects on CHF by expanding the vein, coronary artery, and small peripheral arteries to reduce blood reflow and thus the cardiac preload, thereby improving myocardial blood supply and the cardiac afterload, respectively. However, few studies have examined whether long-acting nitrates can inhibit excessive activation of the SNS and RAS by regulating the expression of AR and angiotensin receptor (ATR) in the kidneys under a condition of CHF. Therefore, in this study, we examined the influence of the administration of long-acting nitrates to a rat model of heart failure induced by myocardial infarction on the expression of AR and ATR in the kidney. These results can lay the foundation for a renal protective effect of long-acting nitrate as a treatment or preventive strategy in patients with CHF.

## Methods

### Experimental animals and the establishment of the CHF model

Clean inbred male Wistar rats (10 weeks old, 250–280 g) were obtained from Beijing Vital River Laboratory Animal Technology Co. Ltd. (Beijing, China). All rats were barrier-housed in the clean animal room with the temperature maintained at 22 ± 3 °C and relative humidity of 50 ± 20% at Capital Medical University affiliated Beijing Anzhen Hospital. The experimental protocol was approved by the institutional ethics committee (permission license: SCXK-2012-0001), and the rats were fed with standardized rat chow and water throughout the experiment.

A total of 90 Wistar rats were randomly divided into three main groups: healthy control group (CTL, *n* = 9), sham-operated group (sham, *n* = 8), and CHF model rats (*n* = 73). The CHF model was induced by ligation of the left anterior descending (LAD) artery. In brief, the rats were anesthetized by intra-abdominal injection of 1% pentobarbital sodium and set on a ventilator for small animals (VT 7–8 ml/kg, respiratory rate 70 per minute, I/E ratio 1:2). Anterior myocardial infarction was created by ligation of the LAD artery near the main pulmonary artery. Four weeks later, echocardiography was performed to evaluate left ventricular (LV) function based on the left ventricular ejection fraction (LVEF); rats with an LVEF ≤45% were considered to have CHF and were included in subsequent experiments [8].

These CHF rats were then randomly divided into the following five treatment groups: CHF model group (CHF, *n* = 9), in which the rats received intragastric administration of saline once a day; low-dose nitrate group (*n* = 9), receiving intragastric administration of 3.6 mg/kg isosorbide-5-mononitrate (IS-5-MN; sustained-release capsules, 50 mg/capsule, UCB Pharma Co. Ltd.) in 2 ml saline once a day; high-dose nitrate group (*n* = 9), receiving intragastric administration of 7.2 mg/kg IS-5-MN in 2 ml saline once a day; positive drug control group (olmesartan; *n* = 9), receiving intragastric administration of 3 mg/kg olmesartan (Olmesartan Medoxomil Tablets, 20 mg/pill, Daiichi Sankyo Co. Ltd.) in 2 ml saline once a day; and high-dose nitrate combined with olmesartan group (nitrate + olmesartan, *n* = 9), receiving intragastric administration with 7.2 mg/kg IS-5-MN and 3 mg/kg olmesartan in 2 ml saline once a day. Sham-operated rats were subject to the same procedure as those in the CHF-induced model but without ligation of the LAD artery. Both the CTL and sham groups received intragastric administration with saline once a day. The treatment lasted for 6 weeks. Rats were anesthetized by intraperitoneal (i.p) injection with sodium pentobarbital (40 mg/kg). And then all blood samples were taken from the abdominal aorta of rats. After blood sampling procedures the animals were euthanized by an injection of sodium pentobarbital (150 mg/kg) administered through the abdominal aorta.

### Echocardiography

Echocardiography was performed to evaluate LV function before treatment and at the end of the experiment (6 weeks later). The rats were anesthetized, and B-mode measurement in the LV short-axis view (papillary muscle level) was performed with a 12-MHz phased array transducer (Vevo 2100 High Resolution Imaging System, Visual Sonics Inc. Toronto, Canada). LVEF was measured and averaged for three consecutive cardiac cycles.

### Plasma renin activity (PRA) and Ang II concentration

At the end of the treatment period, blood samples were collected from the abdominal aorta, and plasma was separated immediately and stored at − 80°C until analysis. For renin activity determination, the plasma was incubated with rabbit angiotensinogen at 37 °C for 60 min and the renin concentration was measured according to the standard protocol of the radioimmunoassay (RIA) kit (IBL, Hamburg, Germany)**,** expressed as nanograms per milliliter per hour.

For Ang II determination, the plasma was incubated with antiserum (anti-rabbit) for 6 h, and then with ^125^I-labeled Ang II for 18 h at 4 °C. Antibody-bound Ang II was separated from the free Ang II using donkey anti-rabbit-coated cellulose. After incubation for 30 min at room temperature and centrifugation at 5000 rpm for 15 min at 4 °C, the concentration of Ang II in each sample was read with the RIA kit according to a prepared standard curve.

### Reverse transcription-polymerase chain reaction (RT-PCR)

The kidneys were removed after blood collection, and specimens of the renal cortex were obtained and frozen in liquid nitrogen at − 80°C until analysis.

Total RNA was extracted from the rat renal cortex by Trizol reagent, and an ultraviolet spectrophotometer was used to detect the concentration of total RNA for each sample. cDNA was synthesized and amplified with a Promega RT-PCR Kit (Madison, WI, USA) according to the manufacturer instructions using sense and antisense oligonucleotide primers synthesized by Bejing SBS Genetech (Bejing, China). To quantify the transcripts obtained by RT-PCR amplification, *Gapdh* was used as an internal standard, and the target mRNA (α_1A_-AR, α_1B_-AR, α_1D_-AR, β_1_-AR, β_2_-AR, β_3_-AR, AT_1_R, and AT_2_R) levels were normalized to that of *Gapdh*. The target genes and *Gapdh* were amplified according to the parameters shown in Table 1.

**Table 1:** Primer subsequence and annealing temperature of α_1_, β adrenergic receptor and Angiotensin II receptor subtypes

Receptor	Primer subsequence	Length of amplified products	Annealing temperature
β_1_-AR	sense:5′-GGGCAACGTTGGTGATCG-3′	213 bp	58 °C
	antisense:5′-CTGGCCGTCACACATAGCAC-3′		
β_2_-AR	sense:5′-GAGACCCTGTGCGTGATTGC-3′	388 bp	58 °C
	antisense: 5′-CCTGCTCCACCTGGCTGAGG-3′		
β_3_-AR	sense:5′-AGTGGGACTCCTCGTAATG-3′	444 bp	59 °C
	antisense: 5′-CGCTTAGCTACGAAC-3’		
α_1A_-AR	sense:5′-CAAGGCCTCAAGTCCGGCCT-3’	156 bp	58 °C
	antisense:5′-CTCTCGAGAAAACTTGAGCAG-3’		
α_1B_-AR	sense:5′-ATCGTGGCCAAGAGGACCAC-3’	287 bp	62 °C
	antisense: 5′-CTCTCGAGAAAACTTGAGCAG-3’		
α_1D_-AR	sense:5′-CGTGTGCTCCTTCTACCTACC-3’	304 bp	58 °C
	antisense:5′-GCACAGGACGAAGACACCCAC-3’		
AT_1_R	sense: 5′-CGTCATCCATGACTGTAAAATTTC-3’	306 bp	53 °C
	antisense: 5′-GGGCATTACATTGCCAGTGTG-3’		
AT_2_R	sense: 5′-GTGTGGGCCTCCAAACCATTGCTA-3’	445 bp	61 °C
	antisense: 5′-TTGCTGCCACCAGCAGAAAG-3’		
GAPDH	sense:5′-TGCACCACCAACTGCTTAGC-3’	196 bp	57 °C
	antisense: 5′-GGCATGGACTGTGGTCATGAG-3’		

*AR* Adrenergic receptor, *ATR* Angiotensin II receptor, *GAPDH* glyceraldehyde-3-phosphate dehydrogenase

RT-PCR products were resolved by electrophoresis on a 1.5% agarose gel (BioRad, USA), and stained with ethidium bromide for visualization on a Bio-rad scanner. Densitometry was used for relative semi-quantitative assessment of expression levels.

### Western blot

Total protein was extracted after homogenizing the rat renal cortex, and the concentration was determined with a bicinchoninic acid protein assay kit. The proteins were separated by 10% sodium dodecyl sulfate-polyacrylamide gel electrophoresis and then transferred to nitrocellulose membranes, which were incubated with the primary antibodies rabbit anti-β_1_-AR (1:200), anti-β_2_-AR (1:200), anti-β_3_-AR (1:200), anti-α_1A_-AR (1:200), anti-α_1B_-AR (1:200), anti-α_1D_-AR (1:200), anti-AT_1_R (1:200), anti-AT_2_R (1:200), and anti-GAPDH (1:200) (all from Santa Cruz Biotechnology, Santa Cruz, CA, USA) at 4 °C overnight, followed by incubation with goat anti-rabbit fluorescent (IRDye-conjugated) secondary antibodies (1:10,000; Rockland Immunochemicals, Gilbertsville, PA, USA) for 2 h at room temperature. The images were quantified by the Odyssey infrared imaging system (LI-COR Biosciences, Lincoln, NE, USA). Levels of proteins were normalized to that of GAPDH.

### Histopathology

The kidneys were perfused with formalin, collected, and fixed in paraffin. Fixed specimens were then cut into 4-μm sections and stained with hematoxylin and eosin following standard procedures. Images were captured with a Nikon Labophot 2 microscope equipped with a Sony CCD-Iris/RGB color video camera attached to a computerized imaging system (Nikon, Japan).

### Statistical analysis

Data are expressed as mean ± standard error of the mean. Differences between groups were analyzed by Student’s *t*-test or one-way analysis of variance, followed by the Newman-Keuls test in GraphPad Prism 5.0 (GraphPad Software Inc., San Diego, CA, USA). *P* < 0.05 was considered statistically significant.

## Results

### Echocardiography results before and after treatment

The echocardiography data are summarized in Table 2. Before treatment, there was no difference in the LVEF between the CTL and sham groups. However, compared to the sham group, the LVEF of all CHF-induced groups was significantly decreased (*P* < 0.01), with no significant differences among the different CHF model groups.

**Table 2:** Echocardiography results before and after therapy

Group	Rats	LVEF/%	
		BT	AT
A	9	72.41 ± 4.93	71.96 ± 4.31
B	8	70.35 ± 7.48	69.37 ± 2.85
C	9	39.46 ± 5.42^a^	36.46 ± 7.78^b^
D	9	39.18 ± 5.75^a^	42.38 ± 7.46
E	9	38.71 ± 6.10^a^	47.54 ± 8.51^c^
F	9	39.17 ± 6.04^a^	49.11 ± 8.34^c^
G	8	38.74 ± 6.25^a^	59.55 ± 4.33^cdef^

^a^*P* < 0.01 vs group B, ^b^*P* < 0.05 vs group B, ^c^*P* < 0.05 vs group C, ^d^*P* < 0.05 vs group D, ^e^*P* < 0.05 vs group E, ^f^*P* < 0.05 vs group F

After treatment, the LVEF of the high-dose nitrate, olmesartan, and nitrate + olmesartan groups significantly increased compared to that of the CHF group, whereas there was no significant effect of treatment with low-dose nitrate. Although the LVEF of the olmesartan group was slightly increased compared to that of the high-dose nitrate group, the difference was not statistically significant. However, the combination of high-dose nitrate and olmesartan had a significant effect on improving the LVEF compared to either treatment alone.

### Plasma PRA and Ang II levels

As shown in Table 3, there was no difference in PRA or Ang II levels between the CTL and sham groups. However, compared with the sham group, the PRA and Ang II plasma levels were significantly increased in the CHF model (*P* < 0.01). However, this increase was significantly alleviated by treatment with high-dose nitrate or olmesartan (*P* < 0.05) and was more strongly improved with their combination (*P* < 0.01); however, low-dose nitrate did not effectively increase the reduction in PRA or Ang II plasma levels. Although olmesartan had a slightly stronger effect on improving PRA and Ang II plasma levels than high-dose nitrate, the difference was not statistically significant. However, their combination had a significant effect on reducing these levels compared to either treatment alone.

**Table 3:** Plasma PRA and Ang II levels ($\overline{x}\pm s$)

group	rats	PRA (μg/L)	Ang II (ng/L)
A	9	4.95 ± 1.42	189.85 ± 60.04
B	8	5.47 ± 1.96	243.98 ± 81.92
C	9	20.14 ± 3.86^a^	531.64 ± 184.13^a^
D	9	17.26 ± 4.38	418.76 ± 85.50
E	9	14.43 ± 4.62^b^	341.60 ± 86.09^b^
F	9	13.91 ± 4.91^b^	330.50 ± 119.22^b^
G	8	7.83 ± 3.87^cdef^	301.48 ± 102.22^c^

^a^*P* < 0.05 versus group B, ^b^*P* < 0.05 versus group C, ^c^*P* < 0.01 versus group C, ^d^*P* < 0.05 vs group D, ^e^*P* < 0.05 vs group E, ^f^*P* < 0.05 vs group F

### Pathological changes of the kidney

**Fig. 1:**
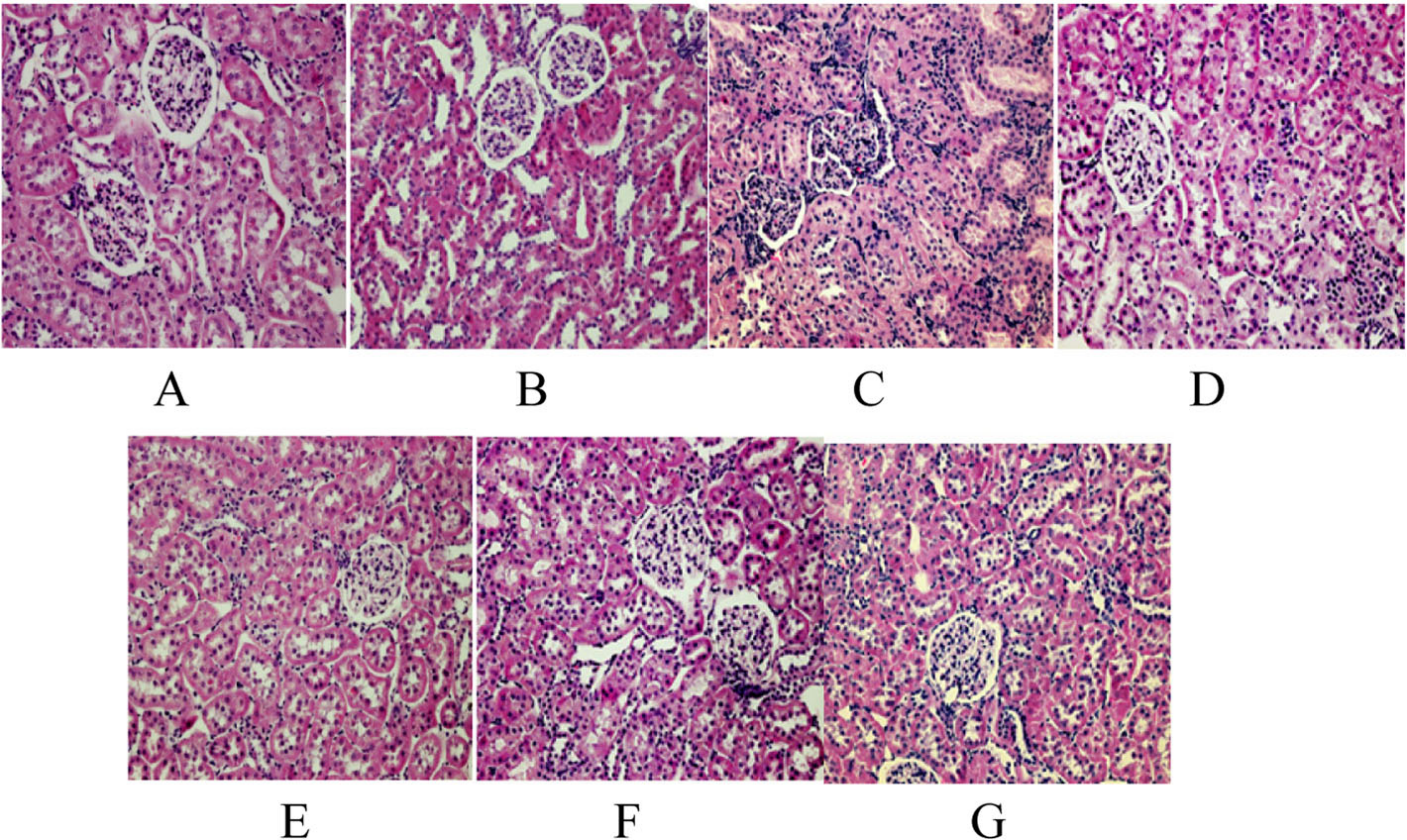
**a**:group A(control group); **b**:group B(sham-operated group); **c**:group C(CHF group); **d**:group D(low-dose nitrate group); **e**:group E(high-dose nitrate group); **f**:group F(positive drug control group); **g**:group G(high-dose nitrate+positive drug group)

The CTL and sham groups both showed a clear structure of the renal glomerulus and tubules with organized glomerular epithelial cells, an intact basement membrane, and no interstitial inflammatory cell infiltration. By contrast, the renal tissue showed significant impairment in the CHF group, including glomerular mesangial area expansion, interstitial inflammatory cell infiltration, partial glomerulus necrosis, and corresponding renal tubular atrophy. However, this inflammatory cell infiltration and tubular necrosis were reduced in all treatment groups to different extents, demonstrating a renal protective effect (Fig. 1).

### Expression of α_1_-, β-AR, and ATR subtypes in the renal cortex

**Fig. 2:**
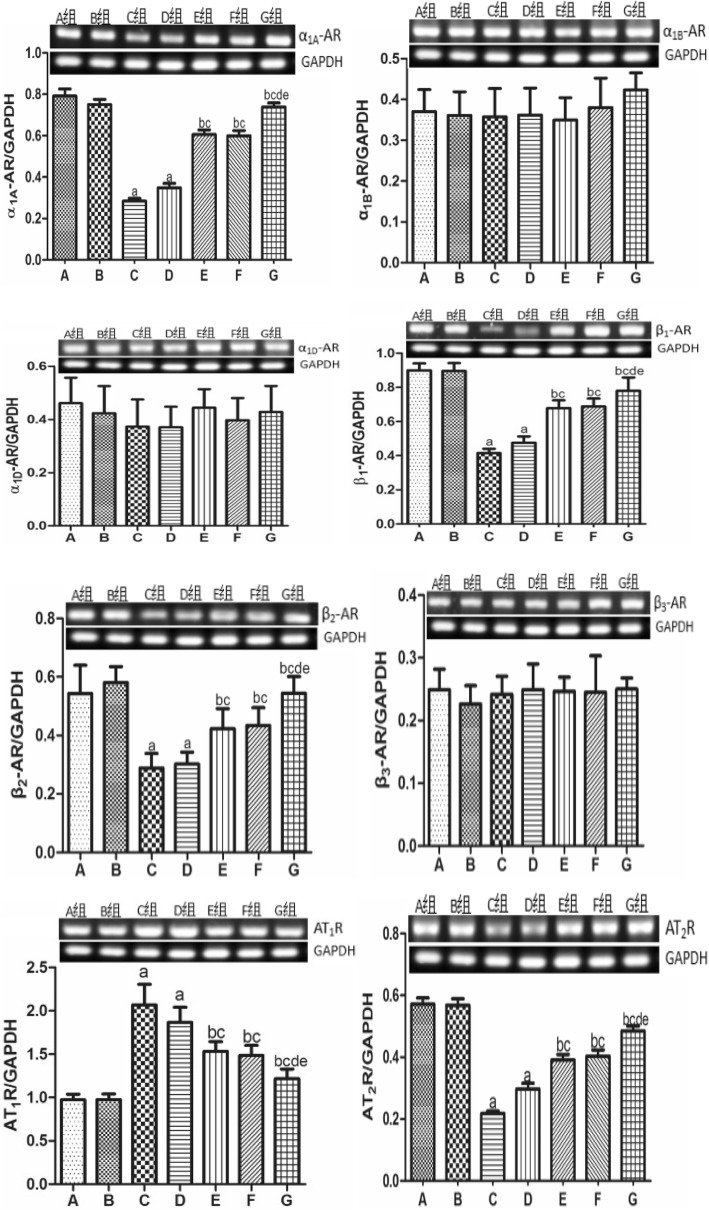
Expression level of α_1_-, β-AR and ATR subtypes mRNA in renal cortex. ^a^*P* < 0.01 vs group B, ^b^*P* < 0.05 vs group C, ^c^*P* < 0.05 vs group D, ^d^*P* < 0.05 vs group E, ^e^*P* < 0.05 vs group F

**Fig. 3:**
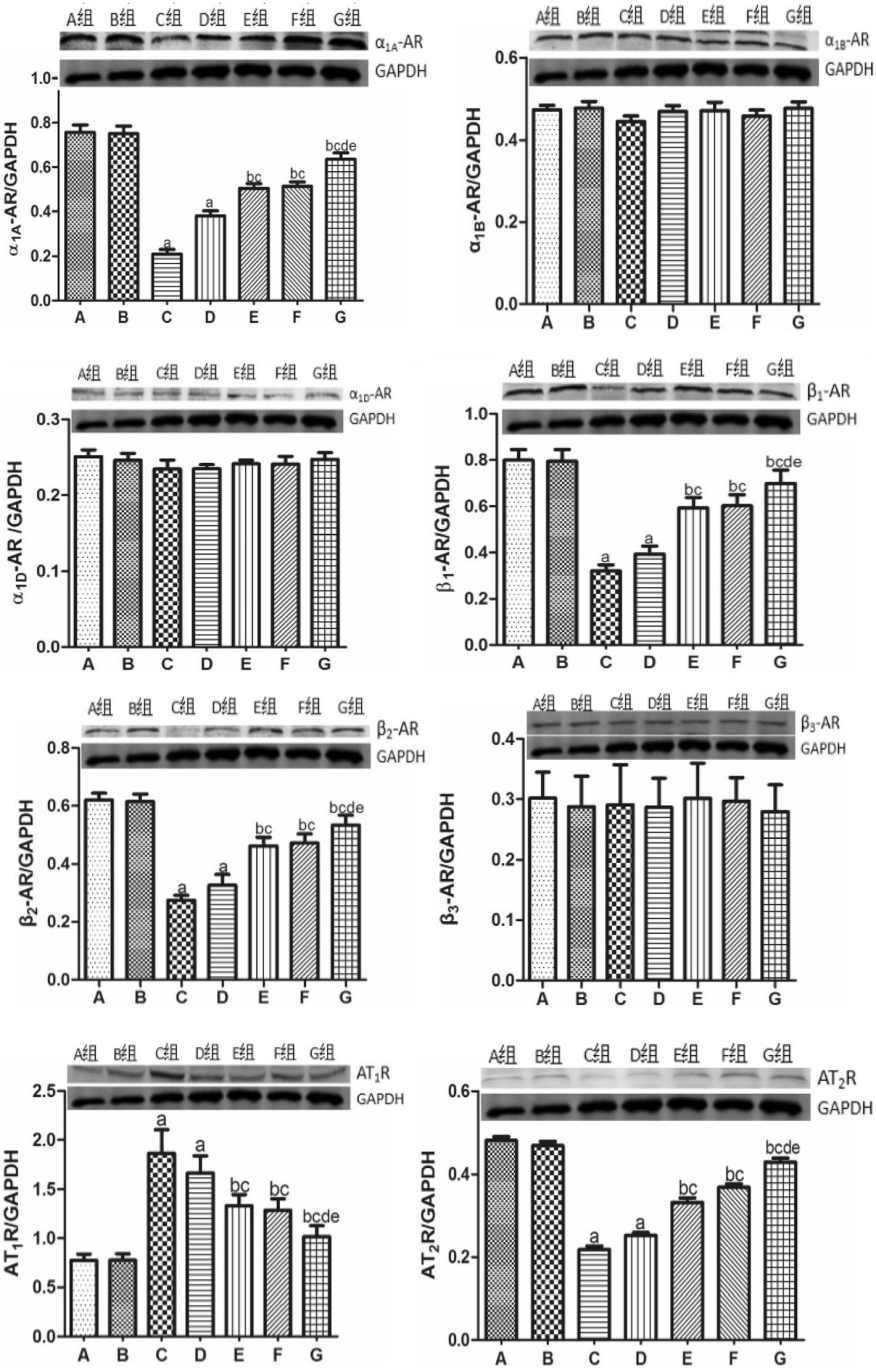
Expression level of α_1_-, β-AR and ATR subtypes in renal cortex. ^a^*P* < 0.01 vs group B, ^b^*P* < 0.05 vs group C, ^c^*P* < 0.05 vs group D, ^d^*P* < 0.05 vs group E, ^e^*P* < 0.05 vs group F

As shown in Figs. 2 and 3, there was no significant difference in the mRNA or protein expression levels of α_1_, β-AR, and ATR subtypes between the sham and CTL groups. Compared with the sham group, the expression of AT_1_R was significantly up-regulated, whereas the expression of α_1A_-AR, β_1_-AR, β_2_-AR, and AT_2_R was down-regulated in the CHF model. Low-dose nitrate treatment did not affect the CHF-induced changes to the levels of α_1_, β-AR, and ATR subtypes, whereas the other treatments significantly reduced the level of AT_1_R and increased the levels of α_1A_-AR, β_1_-AR, β_2_-AR, and AT_2_R. Consistent with the other findings, olmesartan had a more significant effect than high-dose nitrate treatment, but the difference was not statistically significant. Furthermore, the combination treatment had a much greater effect than either treatment alone. There was no significant difference in the expression levels of α_1B_-AR, α_1D_-AR, and β_3_-AR among groups.

## Discussion

Kidneys are vital organs to maintain the body’s blood pressure, as well as the water and electrolyte balance, and the SNS and RAS regulate their physiological functions. After heart failure develops, SNS is activated and the release of catecholamine increases.

In this experiment, the renal tissue showed significant impairment in the CHF group. Inflammatory cell infiltration and tubular necrosis were reduced in all treatment groups to different extents, demonstrating a renal protective effect. However, due to the original design of the study, no quantitative analysis of the histology was performed. We only described the pathological changes under the optical microscope. As the study was completed some time ago and some pathological specimen had been damaged, quantitative analysis of histology could not be performed retrospectively.

In our study, a CHF model was established by ligation of the LV, resulting in a decrease of the LVEF, and an increase of plasma PRA and Ang II levels, indicating excessive activation of the RAS and confirming the successful establishment of the model [9]. However, after treatment of long-acting nitrate, the plasma PRA and Ang II levels decreased. This is likely attributed to a reduction of the venous return and cardiac preload, thereby improving coronary blood supply, and reducing cardiac afterload through expansion of the vein, coronary arteries, and small arteries, respectively.

Moreover, we found that CHF significantly downregulated α_1A_-AR, but did not affect the expression of the other subtypes α_1B_-AR and α_1D_-AR. The result is consistent with previous findings [5, 6], suggesting α_1A_-AR internalization resulting from persistent activation of the SNS in a condition of heart failure, and further weakens its regulatory effect on renal hemodynamics, while RAS activation inhibits the down-regulation of α_1D_-AR and maintains α_1B_-AR at a relatively low baseline expression level. Such receptor-mediated regulation has been shown to play an essential role in maintaining renal perfusion and normal renal function [10, 11]. The up-regulation of α_1A_-AR expression induced by long-acting nitrate treatment may indicate that nitrate improved cardiac function and inhibited excessive activation of the local renal SNS, which then normalized AR expression under the pathological condition to restore its renal protective effect.

There are three β-AR subtypes in the kidney tissue: β_1_-, β_2_-, and β_3_-AR. Stimulating β_1_-AR and β_2_-AR could mediate dilation of the renal arteries and glomerular mesangial cells to regulate blood flow and the glomerular filtration rate (GFR), increase sodium and chloride reabsorption, and increase the secretion of renin and erythropoietin. Besides, β_1_-AR plays a dominant role in the regulation of renal function [3, 12–14]. β_3_-AR can activate nitric oxide synthase (NOS) and promote NO release, and thus cause dilation of the glomerular afferent arteries to adjust the renal perfusion rate [10].

Our results showed that β_1_- and β_2_-AR expression in the heart failure model group was significantly down-regulated, which was consistent with the findings of Fung et al. [12]. One reason for this change could be that stimulation of the SNS increases β-AR kinase activity, leading to down-regulation and desensitization of β-AR. In turn, these receptors can affect renal perfusion and GFR to cause renal damage. This decrease of β_1_- and β_2_-AR expression in the kidney was reversed after nitrate therapy, suggesting that local SNS activity decreases in the kidney after heart function improves so that the receptor expression reverts to the average level under the pathological condition [12]. By contrast, β_3_-AR in the kidney tissue showed a relatively low baseline expression level, and no significant changes were found among the groups.

AT_1_R and AT_2_R are the two ATR subtypes expressed in the renal vasculature and renal tubules [15]. Binding of Ang II to AT_1_R causes renal vasoconstriction, promotes renal sodium and water reabsorption, and enhances cell proliferation, whereas binding of Ang II to AT_2_R can increase NO release, which further contributes to renal vessel dilation, increasing sodium and water excretion, and inhibiting cell proliferation and hypertrophy [16, 17].

In this study, AT_1_R expression was markedly up-regulated in the heart failure group, while AT_2_R expression was significantly down-regulated, suggesting changes of ATR expression levels in the kidney during CHF, which could contribute to renal interstitial edema, inflammatory cell infiltration, glomerular fibrosis, and tubular necrosis, ultimately affecting renal perfusion and reserve function [18, 19]. AT_1_R expression was down-regulated while AT_2_R was up-regulated after nitrate therapy, which may be related to the fact that nitrates can cause renal vascular relaxation, increase renal perfusion, and inhibit RAS activation. Up-regulation of AT_2_R can suppress the expression of AT_1_R, thereby the receptor adjustment protected against impaired renal function during CHF [20, 21].

As a selective AT_1_R antagonist, olmesartan can inhibit RAS activation, and reduce plasma PRA and Ang II levels as well as the renal vascular sensitivity to catecholamines [1, 14]. In contrast to other AT_1_R antagonists, olmesartan has an inverse activating effect when binding to AT_1_R, and thus inactivate AT_1_R and alleviate damage to the kidney induced by Ang II [22, 23]. Moreover, the combination of Ang II and AT_2_R plays a role in physiological renal protection [24].

Compared with monotherapy, high doses of nitrates combined with olmesartan had a more significant effect on improving cardiac function, reducing PRA and Ang II levels, and normalized the receptor expression level, namely by lowering the expression level of AT_1_R and increasing the expression levels of α_1A-_, β_1_-, β_2_-AR, and AT_2_R. These results may relate to the fact that olmesartan blocks AT_1_R and promotes the rise of the tissue endothelial NOS/inducible NOS level and NO synthesis, and by inducing the up-regulation of AT_2_R expression. Thus, olmesartan elevates endothelial NOS levels and increases the bioavailability of NO, which further enhances the vasodilating effect of nitrates [25, 26]. In this study, regulation of receptor expression by high-dose nitrates was significantly superior to that of the low-dose group, which may indicate that large doses of nitrates are required to expand renal vessels more sufficiently.

During CHF, the plasma Ang II level and catecholamine secretion rise, and the sympathetic nerve is activated so that the RAS and SNS interaction increases, resulting in changes of AR and ATR expression in the kidney. Previous studies have shown that Ang II acts on AT_1_R to down-regulate the expression of α_1_-AR, and also phosphorylates and desensitizes β_1_-AR through the PLC/PKC/c-src/PI3K pathway, eventually deteriorating renal function in heart failure [6, 14]. After nitrate therapy, the receptors were inversely regulated, among which β_3_-AR promotes the synthesis of NO by activating NOS, thus mediating renal vasodilation, resulting in reduced AT_1_R expression [27, 28]. This indicates that nitrate can impact the interaction between AR and ATR subtypes, and can improve renal perfusion, inhibit the abnormal proliferation of mesangial cells, reduce interstitial edema and inflammatory cell infiltration, postpone the progression of glomerular fibrosis and tubular necrosis, and finally protect renal function.

## Conclusions

Overall, our results demonstrate that application of long-acting nitrates can, through the interaction between SNS and RAS, inversely regulate the expression of AR and ATR subtypes in the CHF kidney to normal levels, thus playing a beneficial role in protecting renal function. Compared with monotherapy, the combination of nitrates and olmesartan has a more significant effect in regulating the expression of AR and ATR subtypes, showing that long-term nitrates and olmesartan have a synergistic effect on protecting renal function.

## Data Availability

The datasets used and/or analyzed during the current study are not publicly available yet, due to privacy concerns. Data are available from the corresponding author on reasonable request.

## Abbreviations

AR: Adrenergic receptor
AR: Adrenoceptors
ATR: Angiotensin receptor
CHF: Chronic heart failure
PRA: Plasma renin activity
RAS: Renin-angiotensin system
SNS: Sympathetic nervous system

## References

[1] Maser RE, Lenhard MJ, Kolm P (2013). Direct renin inhibition improves parasympathetic function in diabetes. Diabetes Obes Metab.

[2] Zhao Q, Huang H, Wang X (2014). Changes of serum neurohormone after renal sympathetic denervation in dogs with pacing-induced heart failure. Int J Clin Exp Med.

[3] de Lucia C, Femminella GD, Gambino G (2014). Adrenal adrenoceptors in heart failure. Front Physiol.

[4] Lymperopoulos A, Rengo G, Koch WJ (2013). Adrenergic nervous system in heart failure pathophysiology and therapy. Circ Res.

[5] Armenia A, Sattar MA, Abdullah NA (2008). Functional subtypes of renal α1-adrenoceptor in diabetic and non-diabetic 2K1C Goldblatt renovascular hypertension. Acta Pharmacol Sin.

[6] Hye Khan MA, Sattar MA, Abdullah NA (2008). Influence of combined hypertension and renal failure on functional a1-adrenoceptor subtypes in the rat kidney. Br J Pharmacol.

[7] Zhao X, Zhang Y, Leander M, et al. Altered expression profile of renaladrenergic receptor in diabetes and its modulation by PPAR agonists. J Diabetes Res. 2014;725634. 10.1155/2014/725634 PMC3977090 24772448

[8] Zhu W, Zhang X, Xiao Y (2009). Echocardiographic evaluation of cardiac function change in heart failure rat model. J Central South Univ (Medical Sciences).

[9] Gomes-Santos IL, Fernandes T, Couto GK (2014). Effects of exercise training on circulating and skeletal muscle renin-angiotensin system in chronic heart failure rats. PLoS One.

[10] Feng MG, Prieto MC, Navar LG (2012). Nebivolol-induced vasodilation of renal afferent arterioles involves β3-adrenergic receptor and nitric oxide synthase activation. Am J Physiol-Renal Physiol.

[11] Yan-fang SS-TL, Jun L (2007). The influence of angiotensin II on positive inotropic effect mediated by cardiac α_1_-adrenergic receptor in rats of different ages. Chinese J Pharmacol Toxicol.

[12] Fung MM, Chen Y, Lipkowitz MS (2009). Adrenergic beta-1 receptor genetic variation predicts longitudinal rate of GFR decline in hypertensive nephrosclerosis. Nephrol Dial Transplant.

[13] Kim SM, Briggs JP, Schnermann J (2012). Convergence of major physiological stimuli for renin release on the Gs-alpha/cyclic adenosine monophosphate signaling pathway [J]. Clin Exp Nephrol.

[14] Abdulla MH, Sattar MA, Abdullah NA (2011). Effect of renal sympathetic nerve on adrenergically and angiotensin II-induced renal vasoconstriction in normal Wistar-Kyoto rats [J]. Ups J Med Sci.

[15] Gao J, Chao J, Parbhu KJK (2012). Ontogeny of angiotensin type 2 and type 1 receptor expression in mice. J Renin-Angiotensin-Aldosterone Syst.

[16] Yu L, Zheng M, Wang W (2010). Developmental changes in AT1 and AT2 receptor-protein expression in rats. J Renin-Angiotensin-Aldosterone Syst.

[17] Eskildsen TV, Jeppesen PL, Schneider M (2013). Angiotensin II regulates micro RNA-132/−212 in hypertensive rats and humans. Int J Mol Sci.

[18] Clayton SC, Haack KKV, Zucker IH (2011). Renal denervation modulates angiotensin receptor expression in the renal cortex of rabbits with chronic heart failure. Am J Physiol-Renal Physiol.

[19] Ivanov M, Mihailović-Stanojević N, Milanović JG (2014). Losartan improved antioxidant defense, renal function and structure of postischemic hypertensive kidney [J]. PLoS One.

[20] Siragy HM (2010). The angiotensin II type 2 receptor and the kidney. J Renin-Angiotensin-Aldosterone Syst.

[21] Padia SH, Carey RM (2013). AT2 receptors: beneficial counter-regulatory role in cardiovascular and renal function. Pflügers Archiv-European J Physiol.

[22] Tsutamoto T, Nishiyama K, Yamaji M (2010). Comparison of the long-term effects of candesartan and olmesartan on plasma angiotensin II and left ventrieular mass index in patients with hypertension. Hypertens Res.

[23] Sezai A, Soma M, Hata M (2011). Article effects of Olmesartan on the renin-angiotensin-aldosterone system for patients with essential hypertension after cardiac surgery— investigation using a candesartan change-over study. Ann Thorac Cardiovasc Surg.

[24] Naito T, Ma LJ, Yang H (2010). Angiotensin type 2 receptor actions contribute to angiotensin type 1 receptor blocker effects on kidney fibrosis. Am J Physiol-Renal Physiol.

[25] Kim HS, No CW, Goo SH (2013). An angiotensin receptor blocker prevents Arrhythmogenic left atrial remodeling in a rat post myocardial infarction induced heart failure model. J Korean Med Sci.

[26] Thai H, Wollmuth J, Goldman S (2003). AT1 receptor blockade improves vasorelaxation in heart failure by up-regulation of endothelial nitric oxide synthase via activation of the AT2 receptor. J Pharmacol Exp Ther.

[27] Pulakat L, Vincent GD, Adam WC (2011). The impact of Overnutrition on insulin metabolic signaling in the heart and the kidney. Cardiorenal Med.

[28] Lu H, Balakrishnan A, Howatt DA (2012). Comparative effects of different modes of rennin angiotensin system inhibition on hypercholesterolaemia-induced atherosclerosis. Br J Pharmacol.

